# CDP-Diacylglycerol Synthetase Coordinates Cell Growth and Fat Storage through Phosphatidylinositol Metabolism and the Insulin Pathway

**DOI:** 10.1371/journal.pgen.1004172

**Published:** 2014-03-06

**Authors:** Yuan Liu, Wei Wang, Guanghou Shui, Xun Huang

**Affiliations:** 1 State Key Laboratory of Molecular Developmental Biology, Institute of Genetics and Developmental Biology, Chinese Academy of Sciences, Beijing, China; 2 University of Chinese Academy of Sciences, Beijing, China; University of California San Francisco, United States of America

## Abstract

During development, animals usually undergo a rapid growth phase followed by a homeostatic stage when growth has ceased. The increase in cell size and number during the growth phase requires a large amount of lipids; while in the static state, excess lipids are usually stored in adipose tissues in preparation for nutrient-limited conditions. How cells coordinate growth and fat storage is not fully understood. Through a genetic screen we identified *Drosophila melanogaster* CDP-diacylglycerol synthetase (CDS/CdsA), which diverts phosphatidic acid from triacylglycerol synthesis to phosphatidylinositol (PI) synthesis and coordinates cell growth and fat storage. Loss of *CdsA* function causes significant accumulation of neutral lipids in many tissues along with reduced cell/organ size. These phenotypes can be traced back to reduced PI levels and, subsequently, low insulin pathway activity. Overexpressing *CdsA* rescues the fat storage and cell growth phenotypes of insulin pathway mutants, suggesting that *CdsA* coordinates cell/tissue growth and lipid storage through the insulin pathway. We also revealed that a DAG-to-PE route mediated by the choline/ethanolamine phosphotransferase Bbc may contribute to the growth of fat cells in *CdsA* RNAi.

## Author Summary

During development, animals undergo a rapid increase in cell size and number, which requires large amounts of lipids, in the form of phospholipids, for the expansion of cell membranes. Once the growth phase ends, excess lipids are usually stored as body fat, in the form of triacylglycerol (TAG), for use when nutrients are limited. How cells coordinate growth and fat storage is not fully understood. By screening for genes that affect lipid storage in the fruitfly *Drosophila* we discovered that the enzyme CDP-diacylglycerol synthetase (CdsA) coordinates cell growth and fat storage. Phospholipids and TAG have a common precursor, phosphatidic acid, which is diverted by CdsA from TAG synthesis to synthesis of the phospholipid phosphatidylinositol (PI). We also uncovered a link between CdsA and the insulin signaling pathway, which plays a major role in regulating cell and tissue growth. CdsA regulates the level of PI, which modulates insulin pathway activity; insulin pathway activity, in turn, influences the level of CdsA. The lipid metabolism pathways and the insulin signaling pathway are conserved in other animals including humans. Our findings may therefore provide further insights into clinically important imbalances in fat storage such as obesity.

## Introduction

**Figure 1:**
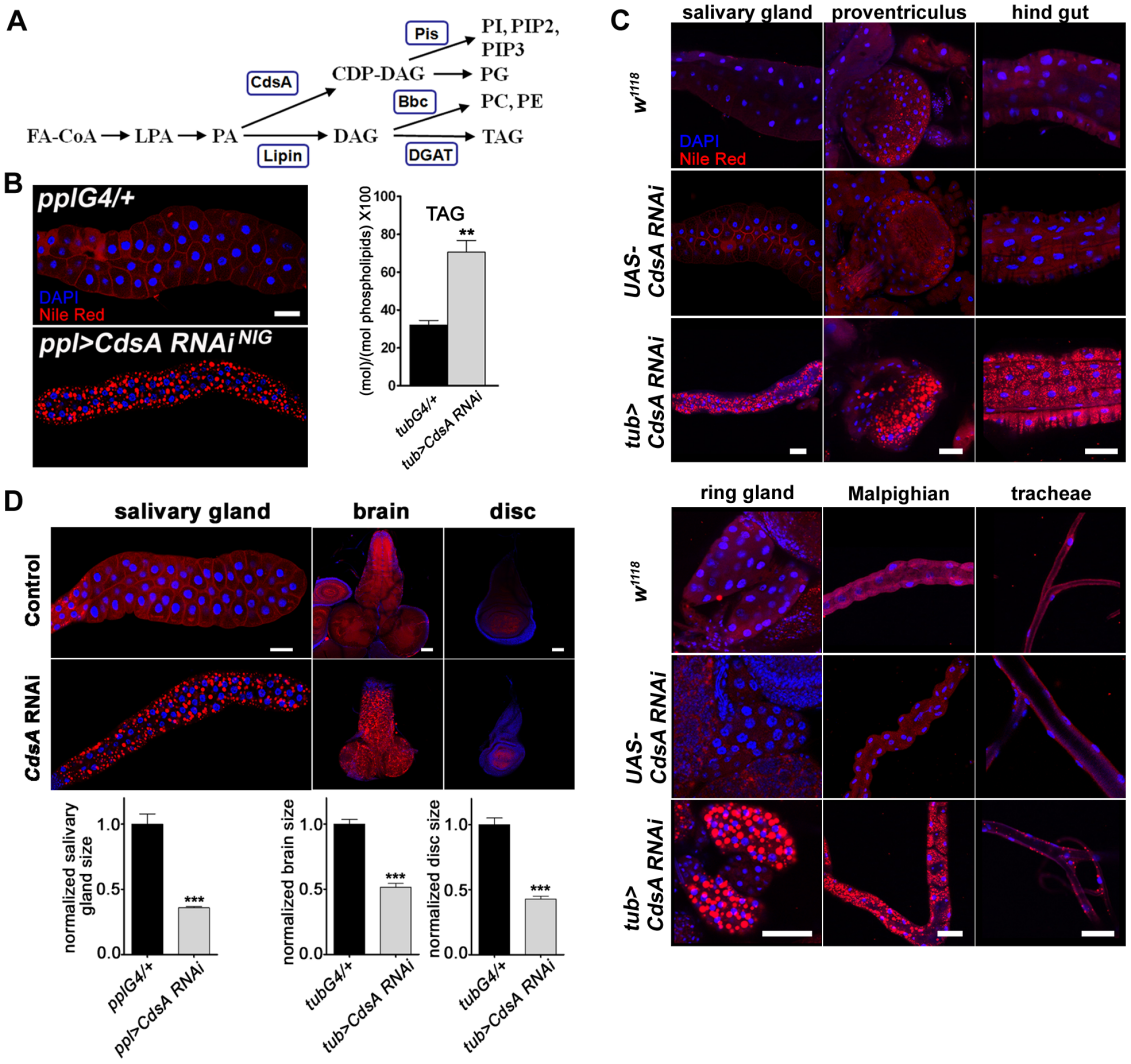
*CdsA* broadly affects fat storage. (A) Simplified schematic of phospholipid and glycerolipid synthesis. FA-CoA: Fatty acyl CoA; LPA: lysophosphatidic acid; PA: phosphatidic acid; DAG: diacylglycerol; TAG: triacylglycerol; CDP-DAG: cytidine diphosphate diacylglycerol; PC: phosphatidylcholine; PE: phosphatidylethanolamine; PI: phosphatidylinositol; PG: phosphatidylglycerol. CdsA adds CTP to PA and generates CDP-DAG; Pis catalyzes the donation of the phosphatidyl group from CDP-DAG to inositol and produces PI, which is the precursor of all PI derivatives, such as PIP2 and PIP3; Bbc synthesizes PC and PE from DAG, which can also be converted to TAG by DGAT. (B) (*left*) RNAi knockdown of *CdsA* in salivary gland causes massive lipid accumulation. *pplG4/+*: the *ppl-Gal4* driver only; *ppl>CdsA RNAi*: *UAS-CdsA RNAi* driven by *ppl-Gal4*. Blue: DAPI staining for nuclei; red: Nile red staining for neutral lipids. (*right*) TAG levels of fat body-removed whole larval samples measured by mass spectrometry. The level of TAG is normalized to total phospholipids. (C) *CdsA* affects fat storage in many tissues. In wandering 3^rd^ instar *tub*>*CdsA RNAi* larvae, massive fat storage in many non-adipose tissues is detected by Nile red staining. Blue: DAPI staining for nuclei; red: Nile red staining for neutral lipids. (D) *CdsA RNAi* reduces salivary gland, brain, and wing disc tissue size. *CdsA* was knocked down with *ppl-Gal4* in salivary gland and *tub-Gal4* in brain and wing disc. Different tissues dissected from wandering 3^rd^ instar larvae of *Gal4* controls and *CdsA RNAi* were stained by Nile red (red) or DAPI (blue). Relative tissue sizes were quantified in multiple samples (salivary gland: n = 8; brain and wing disc: n = 10) based on the area occupied. Scale bar (B, C, D): 50 µm.

During development, animals grow rapidly by both cell proliferation and cell growth. Cell growth is a heavily energy-dependent process and requires large amounts of phospholipids for expansion of cellular membranes and other cellular needs. Fat storage on the other hand is an energy-saving process which stores neutral lipids in the form of triacylglycerol (TAG) for utilization under nutrient-limited conditions such as starvation. In the *de novo* biosynthetic pathway, fatty acyl-CoA is utilized either for TAG synthesis or for phospholipid production (Figure 1A), so it is reasonable to propose that growth and fat storage might be balanced during normal development [1]. Indeed, numerous observations support such a balance. For example, *Caenorhabditis elegans Tor* mutants grow slowly and exhibit excess fat storage [2]. *Drosophila melanogaster* insulin pathway *chico* mutants are less than half the size of wild type, but show an almost 2-fold increase in lipid levels [3].

The mechanisms that coordinate cell growth and neutral lipid storage are largely unknown and many intriguing questions remain to be addressed. During development, animals usually have an initial phase of rapid growth, which involves expansion of cell number and size, followed by a homeostatic state, when growth ceases and excess fat is stored in adipose tissue. How is the balance between cell growth and neutral lipid storage regulated in these two different developmental stages? How is the balance regulated in different tissues or cells? For example, how is the balance regulated in the adipocyte, since it both grows large and stores fat? In some disease states, ectopic lipid accumulation in non-adipose tissues such as muscle, pancreas, and liver is often observed [4]. How is the balance regulated in non-adipose tissues? Answering these questions would definitely lead to a significant advance of our knowledge in the fields of both fat storage and cell growth.

The insulin pathway is a conserved signaling pathway that is essential for cell growth in response to nutrient conditions [5]–[9]. The core components of the insulin pathway include the insulin receptor (InR), insulin receptor substrates (IRS), phosphatidylinositol 3-kinase (PI3K), the protein kinase Akt, and the transcription factor FOXO. PI3K, which generates phosphatidylinositol 3,4,5-trisphosphate (PIP3) from phosphatidylinositol 4,5-bisphosphate (PIP2), and PTEN, the phosphoprotein phosphatase that converts PIP3 to PIP2, are positive and negative regulators of the insulin pathway, respectively [10], [11]. PIP4K, which generates PIP2 from phosphatidylinositol 5-phosphate (PIP) also positively regulates insulin pathway activity [12]. Therefore, various enzymes that affect the level of PIP3 provide an important layer of regulation of insulin pathway activity. Recently, several novel components of the insulin pathway were identified, including miRNAs (miR-8 and miR-14) and secreted proteins (Upd and SDR) [13]–[16].

It is well known that the insulin pathway regulates lipid homeostasis and couples nutritional conditions with systemic growth and metabolism. Besides inhibiting lipolysis, it also promotes fatty acid synthesis through activating the expression of different target genes, including acetyl-CoA carboxylase (ACC), fatty acid synthase (FAS), and sterol regulatory element binding protein (SREBP) [17]–[19]. Recently acyl-CoA synthetase (ACS)/Pudgy has been identified as a direct target of FOXO [20]. Since many of these regulators have been studied during the homeostatic stage, it is not known whether they contribute to the balance of cell growth and neutral lipid storage during the developmental stage. Furthermore, additional regulators or targets of the insulin pathway in balancing cell growth and fat storage remain to be identified.

With its exquisite genetic tools and evolutionarily conserved metabolic pathways, *Drosophila melanogaster* has been accepted as a model for studying lipid metabolism [21]–[24]. Through continuous feeding, *Drosophila* larvae grow rapidly with a nearly 200-fold increase in body mass during the 4-day larval stage. During this time most tissues, such as brain, salivary gland, and imaginal discs, store very little neutral lipid, while the fat body accumulates large amounts of fat [23]. The fat body is a specialized energy storage organ, equivalent to the vertebrate adipose tissue, and most of the dietary fats and *de novo*-synthesized fats from the intestine are transported to the fat body via lipoproteins in the hemolymph [25]. As in mammals, many questions remain to be answered in *Drosophila*. How do *Drosophila* larvae coordinate cell growth and neutral lipid storage in non-adipose tissues? What mechanisms are used in the fat body to balance the storage of fat along with cell growth?

In this study, we identified that *Drosophila* CDP-diacylglycerol synthetase, CdsA, coordinates cell growth and neutral lipid storage through phosphatidylinositol (PI) metabolism and the insulin pathway. We also found that when *CdsA* is defective, a DAG-to-PE route mediated by the choline/ethanolamine phosphotransferase Bbc may contribute to the growth of fat cells.

## Results

### *CdsA* broadly affects fat storage

We previously reported that mutants of *dSeipin*, the *Drosophila* homolog of human lipodystrophy gene *Seipin* which is important for fat storage and lipid droplet size [26], [27], exhibit ectopic lipid droplets in salivary glands, which can be stained by the neutral lipid dyes Nile red or Bodipy [28]. The *Drosophila* salivary gland is large and easy to manipulate and has been previously used as an *in vivo* system to study cell death and autophagy, cell growth and proliferation, and signal transduction, for example in Hedgehog signaling [29]. Hence, we investigated neutral lipid storage regulation in salivary glands by screening for genes which, when knocked down or overexpressed, caused an ectopic lipid droplet phenotype in salivary gland. We used *ppl-Gal4*, which drives strong gene expression in both larval salivary gland and fat body. The strongest ectopic lipid storage phenotype was caused by *CdsA* RNAi (Figure 1B). The phenotype was also verified with an independent *CdsA* RNAi line (Figure S1A). In addition, *CdsA^1^*, a weak allele of *CdsA*, was previously found to have a *dSeipin*-like ectopic lipid storage phenotype in the salivary gland [28], demonstrating that *CdsA* affects fat storage. CdsA is the sole *Drosophila* CDP diglyceride synthetase (Cds), and diverts phosphatidic acid (PA) from TAG synthesis to the synthesis of cytidine diphosphate diacylglycerol (CDP-DAG), the precursor of two phospholipids, PI and phosphatidylglycerol (PG) (Figure 1A).

According to results from the *Drosophila* gene expression database FlyAtlas (http://www.flyatlas.org), *CdsA* is widely expressed in different larval tissues. In adults, *CdsA* is highly enriched in the eye and is important for the phototransduction pathway [30]. We confirmed the larval expression profile with Q-RT-PCR (Figure S1B) and a Lac-Z enhancer trap line. The Lac-Z signal was detected in many places, including salivary gland, fat body, proventriculus, hind gut, brain, muscle, Malpighian tubules, and tracheal tubes (Figure S1C). The broad expression of *CdsA* and the strong salivary gland fat storage phenotype of *CdsA RNAi* provide a unique opportunity to address whether other non-adipose tissues have the same capacity as salivary glands to store excess fat.

When we drove *UAS-CdsA RNAi* transgene expression using the ubiquitous *tub-Gal4* driver, a strong, fully penetrant phenotype was observed. The level of *CdsA* transcripts was reduced to about 40% with RNAi. The *tub>CdsA RNAi* larvae accumulated excess fat in many non-adipose tissues, including salivary gland, proventriculus, hind gut, Malpighian tubules, and trachea (Figure 1C). Among the tissues examined, the most prominent lipid storage phenotype was found in salivary gland and prothoracic gland, both of which have polyploid cells. The excess lipid accumulation phenotype of *tub>CdsA RNAi* was reproduced using various tissue-specific *Gal4* lines, suggesting that *CdsA* acts tissue-autonomously (data not shown). To direct measure the accumulation of TAG in non-adipose tissues, we measured the level of TAG from the fat body-removed whole larval samples. Compared to control, *tub>CdsA RNAi* animals have much higher level of TAG, consistent with the Nile red staining results (Figure 1B). Together, these results indicate that although the fat body is the main energy reservoir for larvae, neutral lipids can still be stored in significant amounts in many non-adipose tissues when CdsA function is lost. However, it remains to be determined whether excess lipid accumulation in these non-adipose tissues can fulfill the energy reservoir function of the fat body, or causes deterioration of these tissues.

### *CdsA* regulates salivary gland fat storage and cell size

Beside the fat storage phenotype, we noticed a significantly reduced organ size phenotype in *tub>CdsA RNAi* larvae. The imaginal discs, salivary gland, and brain are all smaller in *tub>CdsA RNAi* larvae than controls (Figure 1D). This phenotype could be due to a decrease in cell size, or cell number, or both. We quantified the cell size and cell number in the salivary gland and found that while the cell number is not changed, the cell size is greatly reduced in *tub>CdsA RNAi* larvae (data not shown). Therefore, reduced cell size is the likely cause of the small organ size in *CdsA RNAi* larvae.

**Figure 2:**
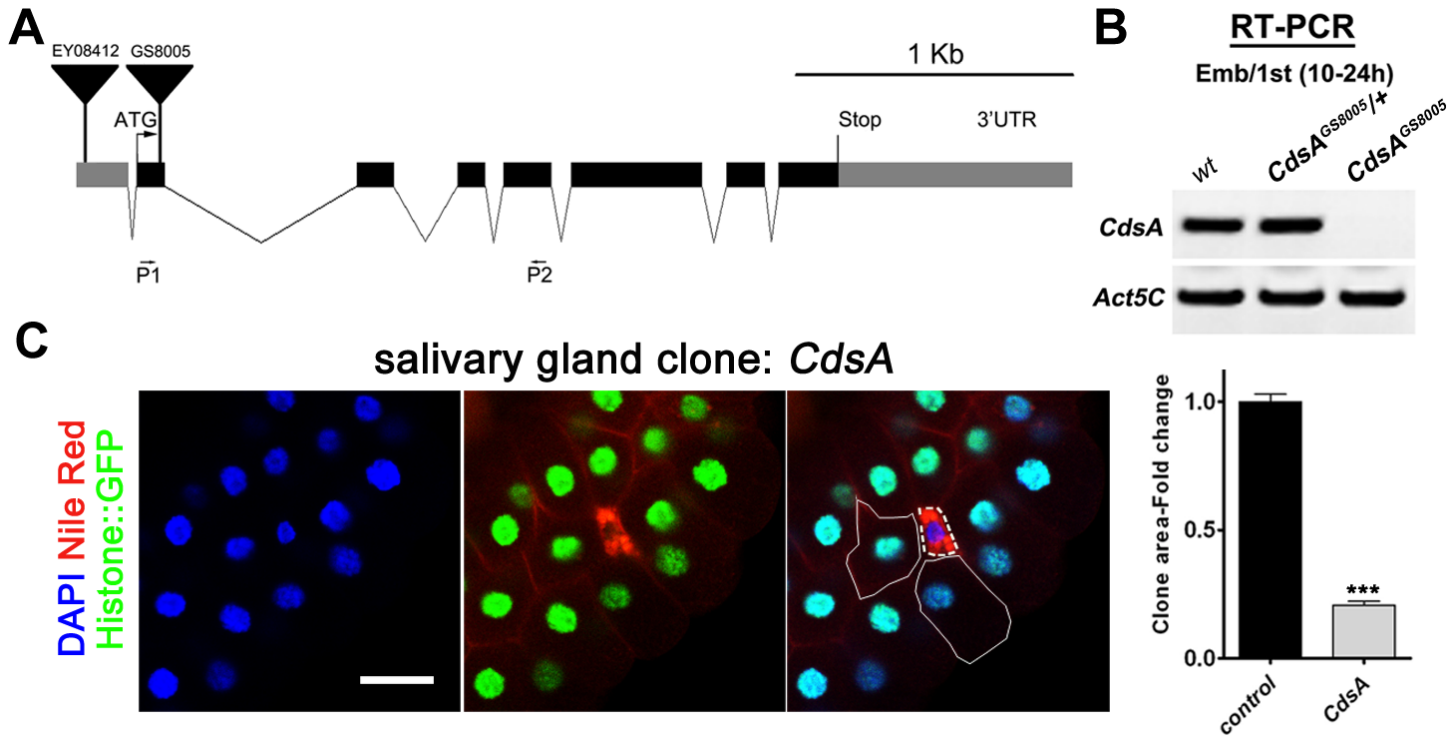
*CdsA* mutations affect salivary gland fat storage and cell size. (A) Schematic representation of the *CdsA* (*CG7962*) genomic locus. The location within *CdsA* of two *P* element insertions, *EY08412* (for overexpression studies) and *GS8005* (for mutant clonal analysis) are shown. P1 and P2: primers for RT-PCR in (B). (B) The *CdsA^GS8005^* allele is likely a null. Embryo/1^st^ instar larval (10–24 hr) RNA of *wt*, trans-heterozygous or homozygous *CdsA^GS8005^* was analyzed by RT-PCR. Note that there is no detectable *CdsA* transcript in *CdsA^GS8005^* mutants. *Act5C* is a control for RT-PCR. (C) A *CdsA* mutant cell (non-GFP, dashed white circle) is small and contains more neutral lipids compared with neighboring control cells (solid white circle). *CdsA* mutant salivary gland clones were induced by flp/FRT-mediated recombination during embryogenesis and visualized in wandering 3^rd^ instar larvae. Blue: DAPI staining for nuclei; red: Nile red staining for neutral lipids; green: Histone::GFP. Histogram: n = 8. Scale bar (C): 50 µm. Error bars (C) represent SEM. (*) P<0.05; (**) P<0.01; (***) P<0.001 (Student's t-test).

To rule out a possible off-target effect of RNAi, we turned to *CdsA* mutants. *CdsA^1^* is a weak and viable allele of *CdsA* and does not cause an obvious small cell size phenotype (data not shown). In the *CdsA^GS8005^* allele, a transposon element inserted into the second exon of *CdsA* disrupts transcription (Figure 2A). *CdsA^GS8005^* animals died at late embryonic to early larval stages. By RT-PCR, *CdsA^GS8005^* is likely a strong loss-of-function or null allele (Figure 2B). We generated *CdsA^GS8005^* mutant clones in the salivary gland. Consistent with the RNAi result, *CdsA^GS8005^* mutant salivary gland cells are smaller in size and accumulate large amounts of fat compared to neighboring control cells (Figure 2C), further demonstrating that *CdsA* functions cell-autonomously. On average, the size of *CdsA^GS8005^* mutant salivary gland cells is only about 20% of wild-type cells. Together, these results suggest that *CdsA* plays a key role in balancing fat storage and cell growth.

### *CdsA* regulates salivary gland cell growth by affecting PI metabolism and insulin pathway activity

**Figure 3:**
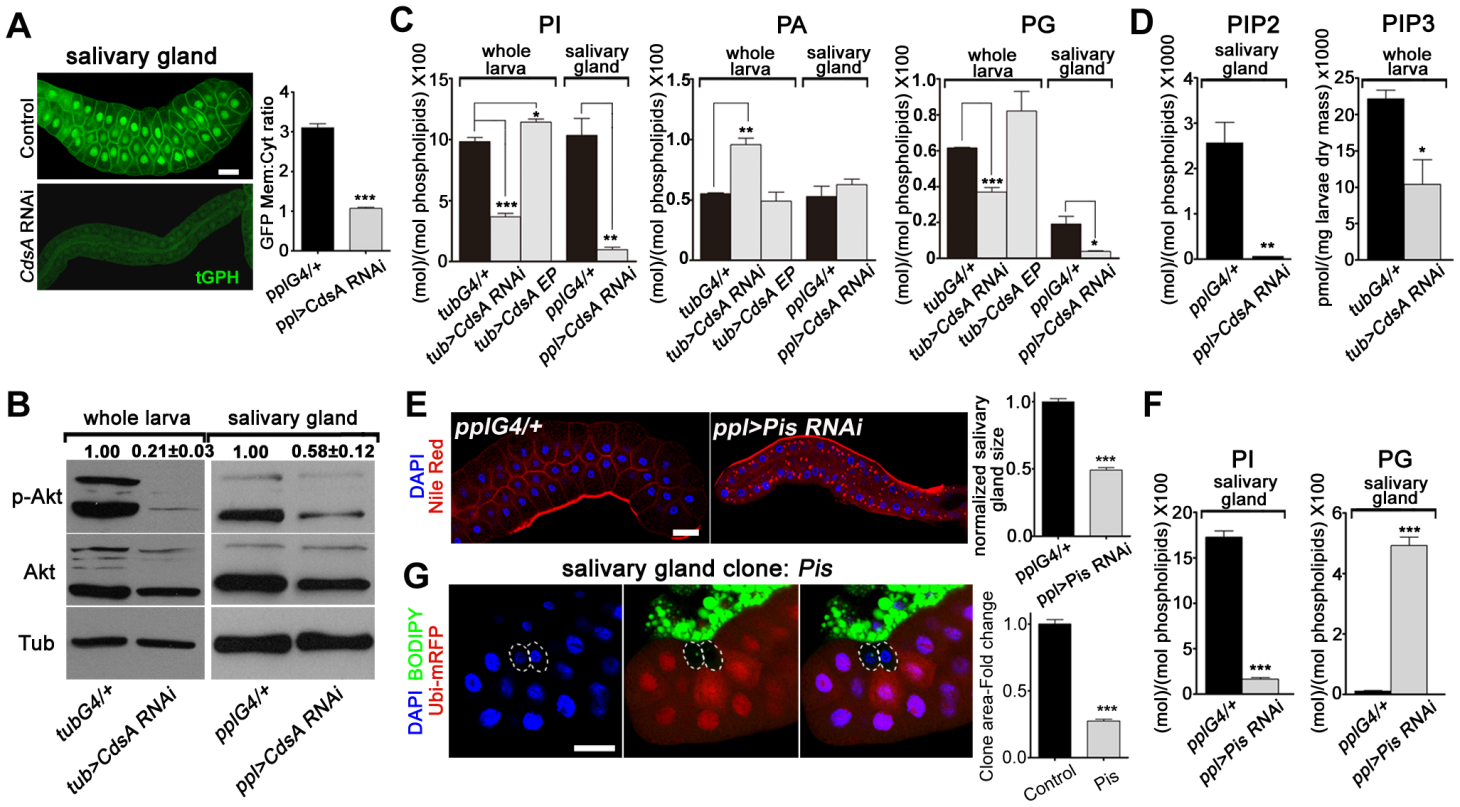
*CdsA RNAi* affects PI metabolism and insulin pathway activity. (A) tGPH reporter assay in salivary gland cells. The cell membrane fluorescence signal is significantly weaker and more diffuse in *CdsA RNAi* than in the *ppl-Gal4* control. Images were taken with the same exposure time. Membrane-to-cytoplasmic GFP intensity ratios were calculated from measurements of mean pixel intensities within equal areas of membrane versus cytoplasm. Histogram: n = 21. (B) Total Akt and phosphorylated Akt (Ser505) levels were detected by western blotting. α-tubulin (Tub) was used as a loading control. Average and standard deviation of relative band intensity ratio of p-Akt *versus* total Akt from three replicates is indicated at the top after normalization. The Western blot result from one experiment is shown here. In whole larva and salivary gland, RNAi of *CdsA* diminished Akt phosphorylation at serine505. (C) Phospholipid levels obtained by lipid profiling of whole larva or salivary gland samples from wandering 3^rd^ instar larvae with the following genotypes: *Gal4* control (*G4/+*), *CdsA RNAi*, and *CdsA* overexpression (*CdsA EP*). *CdsA* was knocked down with *ppl-Gal4* in salivary gland and *tub-Gal4* in whole larvae. Assays were done in triplicate. Note that the PI level in the *CdsA RNAi* salivary gland sample is less than 10% of that in the *Gal4* control. The levels of PI, PA, and PG are normalized to total phospholipids. (D) PIP2 and PIP3 levels measured by mass spectrometry and ELISA kit, respectively. Assays were done at least in triplicate. The level of PIP2 is normalized to total phospholipids. The level of PIP3 is normalized to dry weigh. (E) Silencing *Pis* reduces salivary gland size and causes accumulation of lipid droplets. Blue: DAPI staining for nuclei; red: Nile red staining for neutral lipids. Histogram: n = 9. (F) Phospholipid levels obtained by profiling of salivary gland samples from control and *Pis RNAi* wandering 3^rd^ instar larvae. Assays were done in triplicate. The levels of PI and PG are normalized to total phospholipids. (G) *Pis* mutant salivary gland cells (marked by the absence of RFP, dashed white circles) are significantly smaller than neighboring wild-type cells. *Pis* mutant salivary gland clones were induced during embryogenesis and visualized in wandering 3^rd^ instar larvae. Blue: DAPI staining for nuclei; green: BODIPY staining for neutral lipids; red: Ubi-mRFP, which marks cytosol. Histogram: n = 11. Scale bar (A, E, G): 50 µm. Error bars (A, C, D, E, F, G) represent SEM. (*) P<0.05; (**) P<0.01; (***) P<0.001 (Student's t-test).

Cell growth depends on nutrient conditions and is well-known to be regulated by the insulin signaling pathway. The activity of the insulin pathway is regulated by the PI-derived lipid PIP3. Since CdsA is a key enzyme involved in the production of PI from PA through CDP-DAG (Figure 1A), and PI is the precursor of PIP, PIP2, and PIP3, we speculated that in *CdsA RNAi* or mutant animals, the total level of PI is reduced, resulting in a reduction in PIP3, which would lead to low insulin pathway activity and defective cell growth. To test this hypothesis, we first examined the level of PIP3 with a PIP3-specific GFP reporter, tGPH (*tubulin*-GFP-Pleckstrin homology), in the salivary gland [10]. GFP is recruited to the plasma membrane by binding to PIP3; therefore, the ratio of plasma membrane to cytosol GFP intensity reflects the relative level of PIP3. In controls, the GFP signal is strong in both plasma membranes and nuclei. In *CdsA RNAi* larvae, the overall GFP intensity is much lower due to unknown reasons and the plasma membrane to cytosol GFP intensity ratio is only 30% of wild type, indicating reduced levels of PIP3 (Figure 3A). To further probe the activity of the insulin pathway in *CdsA RNAi*, we also determined the level of phosphorylation of residue S505 of the protein kinase Akt (P-Akt^S505^, equivalent to S473 in mammalian Akt), which has often been used as an indicator of insulin pathway activity. The P-Akt^S505^ level is significantly decreased in *CdsA RNAi* whole larvae and salivary glands (Figure 3B).

To obtain direct evidence of reduced PI levels, we quantified PI lipids in whole larval and salivary gland samples using mass spectrometry. We found that in both whole larvae and salivary glands, the total level of PI is greatly reduced in *CdsA RNAi* samples compared to controls and the level of PI in salivary glands is reduced more than ten-fold (Figure 3C). *CdsA RNAi* also causes a reduction in the total level of PG, accompanied by an increase in PA (Figure 3C). In addition, ubiquitous overexpression of *CdsA* slightly increases the levels of PI and PG in whole larval samples (Figure 3C). These data are consistent with the role of CdsA in catalyzing the conversion of PA to CDP-DAG, the precursor of PI and PG. Besides, compared to measurable amount of PIP2 in control, the level of PIP2 in *CdsA RNAi* salivary gland samples is below the threshold level detected by mass spectrometry (Figure 3D). Moreover, likely due to the small size of the salivary glands, we were unable to measure the abundance of PIP3 in both control and *CdsA RNAi* samples with mass spectrometry. Instead, we measured the level of PIP3 in whole larval samples using an ELISA method. In *CdsA* knockdown larvae, the PIP3 level is reduced to less than half of the control (Figure 3D). Taken together, our results indicate that *CdsA* influences insulin pathway activity and PI metabolism in the salivary gland.

These results predict that blocking *de novo* PI synthesis in the salivary gland should mimic the *CdsA* mutant clone phenotype in affecting salivary gland cell growth. Phosphatidylinositol synthase (Pis) is the only enzyme which synthesizes PI from CDP-DAG and inositol (Figure 1C). Similar to *CdsA RNAi* salivary glands, *Pis RNAi* salivary glands are small and accumulate lipid droplets (Figure 3E). Similar to *CdsA RNAi*, the level of PI in salivary glands is greatly reduced in *Pis RNAi* samples compared to controls (Figure 3F). On the other hand, the level of PG is significantly increased in *Pis RNAi* (Figure 3F). We further examined *Pis^1^*, a likely null mutant of *Pis* [31]. The *Pis^1^* mutation is lethal, like *CdsA^GS8005^*, so we generated *Pis^1^* mutant clones. Interestingly, *Pis^1^* mutant salivary gland cells do not accumulate large numbers of lipid droplets (Figure 3G), suggesting that *Pis* mutations likely increase the level of CDP-DAG, which is converted to PG, without significantly elevating the level of PA. Similar to *CdsA^GS8005^* mutant salivary gland cells, *Pis^1^* mutant salivary gland cells are significantly smaller than wild-type cells (Figure 3G). On average, the size of *Pis^1^* mutant salivary gland cells is only about 25% of wild-type cells. The reduction of cell size in *CdsA* and *Pis* mutants is not as strong as in insulin pathway mutants, where salivary gland cell size can be reduced by over 90% [10], [32], suggesting that PI from other resources, such as uptaken externally or inherited from mother cells, may partly contribute to salivary gland cell growth. Taking these results together, we conclude that *CdsA* regulates salivary gland cell growth by affecting PI metabolism and insulin pathway activity.

### The insulin pathway genetically interacts with *CdsA* and affects *CdsA* transcription levels

**Figure 4:**
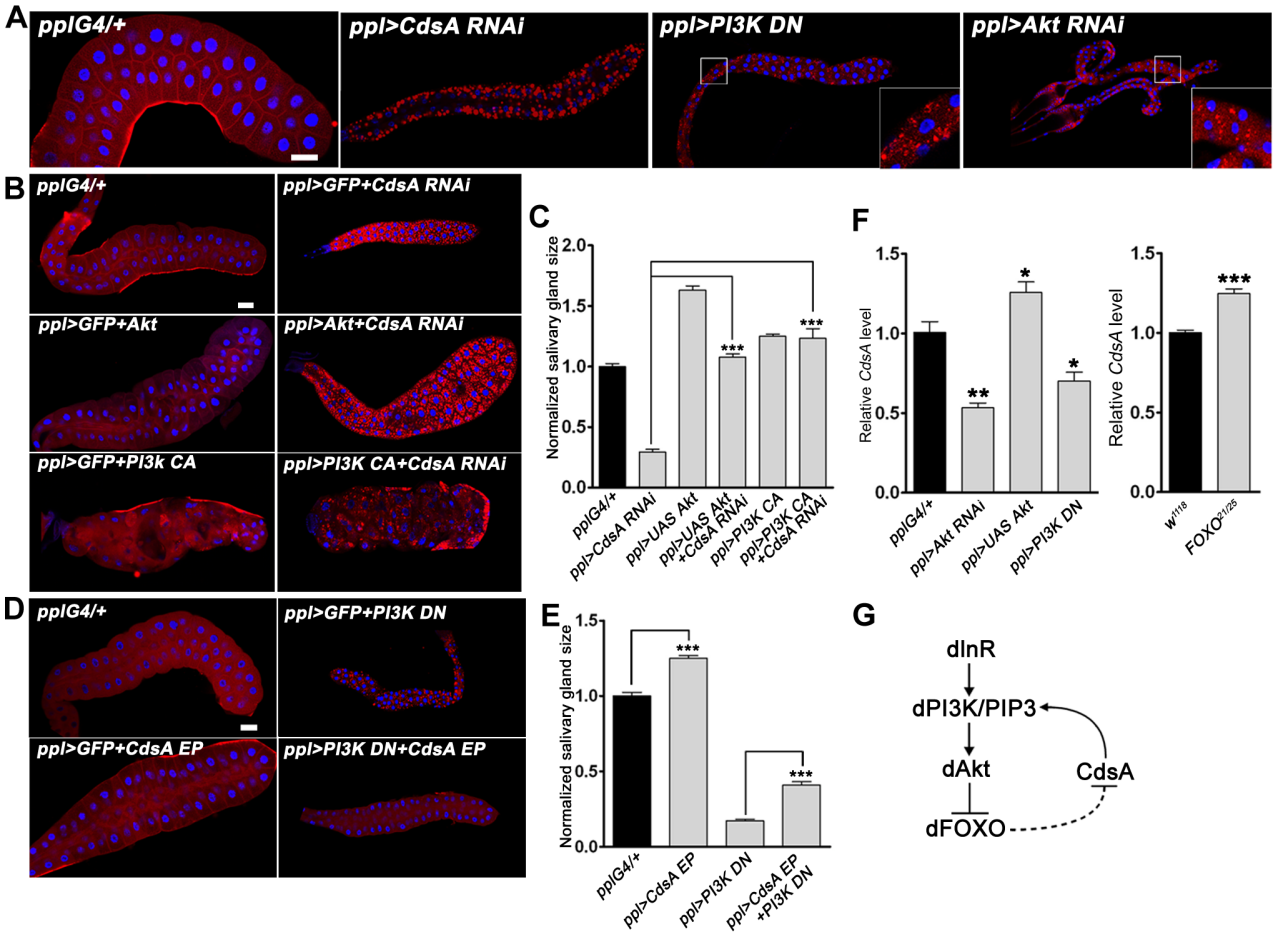
The insulin pathway genetically interacts with *CdsA* and affects the transcription level of *CdsA*. (A) Perturbation of insulin pathway activity affects salivary gland size and fat storage. Expression of dominant-negative *PI3K* (*PI3K DN*) or knockdown of *Akt* by RNAi leads to lipid accumulation in salivary glands of dramatically reduced size, reminiscent of *CdsA RNAi*. Blue: DAPI staining for nuclei; red: Nile red staining for neutral lipids. (B, C) Elevation of insulin pathway activity by overexpressing *Akt* or by expressing constitutively active *PI3K (PI3K CA)* fully rescues the small salivary gland size phenotype of *CdsA RNAi*. Note that the massive fat accumulation in *CdsA RNAi* salivary gland is also partially rescued by *PI3K CA* expression. Blue: DAPI staining for nuclei; red: Nile red staining for neutral lipids. (D, E) Overexpressing *CdsA* partially but significantly rescues the small salivary gland phenotype of *PI3K DN*. Note that the lipid accumulation in *PI3K DN* can be fully suppressed by *CdsA* overexpression. Blue: DAPI staining for nuclei; red: Nile red staining for neutral lipids. (F) The insulin pathway positively regulates *CdsA* expression. Relative *CdsA* mRNA levels were quantified by qRT-PCR on dissected salivary glands from wandering 3^rd^ instar larvae of each genotype. Measurements were made in triplicate. (G) The positive feedback loop between the insulin pathway and *CdsA*. CdsA affects insulin pathway activity by regulating PI/PIP3 levels, while the insulin pathway regulates the *CdsA* transcription level. Histogram (*C*, *E*): n≥8 for each genotype. Scale bar (A, B, D): 50 µm. Error bars (C, E, F) represent SEM. (*) P<0.05; (**) P<0.01; (***) P<0.001 (Student's t-test).

To further reveal the connections between *CdsA*, PI metabolism, and the insulin pathway, we turned to genetic interaction assays. In the salivary gland genetic screen, we identified two insulin pathway components, *PI3K* and *Akt*, in addition to *CdsA*. Knockdown of *PI3K* by dominant-negative *PI3K* (*PI3K DN*) or of *Akt* by RNAi leads to small cell size and ectopic lipid storage phenotypes in the salivary gland, although the lipid storage phenotype is weaker than that of *CdsA RNAi* (Figure 4A and S2A). Since both *CdsA RNAi* and reduced insulin pathway activity have the same phenotypes in the salivary gland, we examined their genetic interaction by gain of insulin pathway activity in *CdsA RNAi* larvae and *vice versa*. We found that overexpressing wild-type *Akt* or constitutively active *PI3K (PI3K CA)* fully suppressed the small cell size phenotype of *CdsA RNAi* (Figure 4B and 4C). Although we can't rule out the possibility that CdsA and insulin pathway act redundantly in a common pathway, these results are consistent with the notion that *CdsA* acts upstream of PI3K in positively regulating insulin pathway activity. Interestingly, the ectopic lipid accumulation phenotype of *CdsA RNAi* is partially rescued by overexpression of *PI3K CA*, but not *Akt* (Figure 4B and S2B).

We then tested whether overexpression of *CdsA* can rescue the cell size and ectopic lipid storage phenotypes caused by impaired insulin pathway activity. On its own, *CdsA* overexpression slightly increases the size of the salivary gland (Figure 4D and 4E). Interestingly, *CdsA* overexpression partially, but significantly, rescued the small cell size phenotype of *PI3K DN* (Figure 4D and 4E). Moreover, the salivary gland ectopic lipid storage phenotype of *PI3K DN* can be fully suppressed by *CdsA* overexpression (Figure 4D and S2B). These results raise the possibility that insulin pathway activity may influence the *CdsA* level.

Since insulin signaling regulates lipid homeostasis mainly at the level of gene transcription and the forkhead box protein dFOXO can transcriptionally activate the upstream target InR via a feedback regulatory loop [33], we asked whether the transcription of *CdsA* is affected by the insulin pathway. We examined the level of *CdsA* under conditions which perturb insulin pathway activity. Compared to controls, *CdsA* transcription levels in *PI3K DN* or *Akt RNAi* salivary glands were reduced significantly to 40–50% as assayed by quantitative RT-PCR (Figure 4F). Conversely, *CdsA* transcription levels slightly increased when *Akt* was overexpressed (Figure 4F). Likewise, loss of FOXO slightly increased the level of *CdsA* (Figure 4F). Taken together, these results pinpoint a positive feedback loop between insulin signaling and *CdsA* in modulating the balance of cell growth and lipid storage. In this positive feedback loop (Figure 4G), *CdsA* positively regulates the activity of the insulin pathway through PI, and the insulin pathway affects the transcription of *CdsA*.

### Fat body cell growth and neutral lipid storage are not affected by *CdsA*

**Figure 5:**
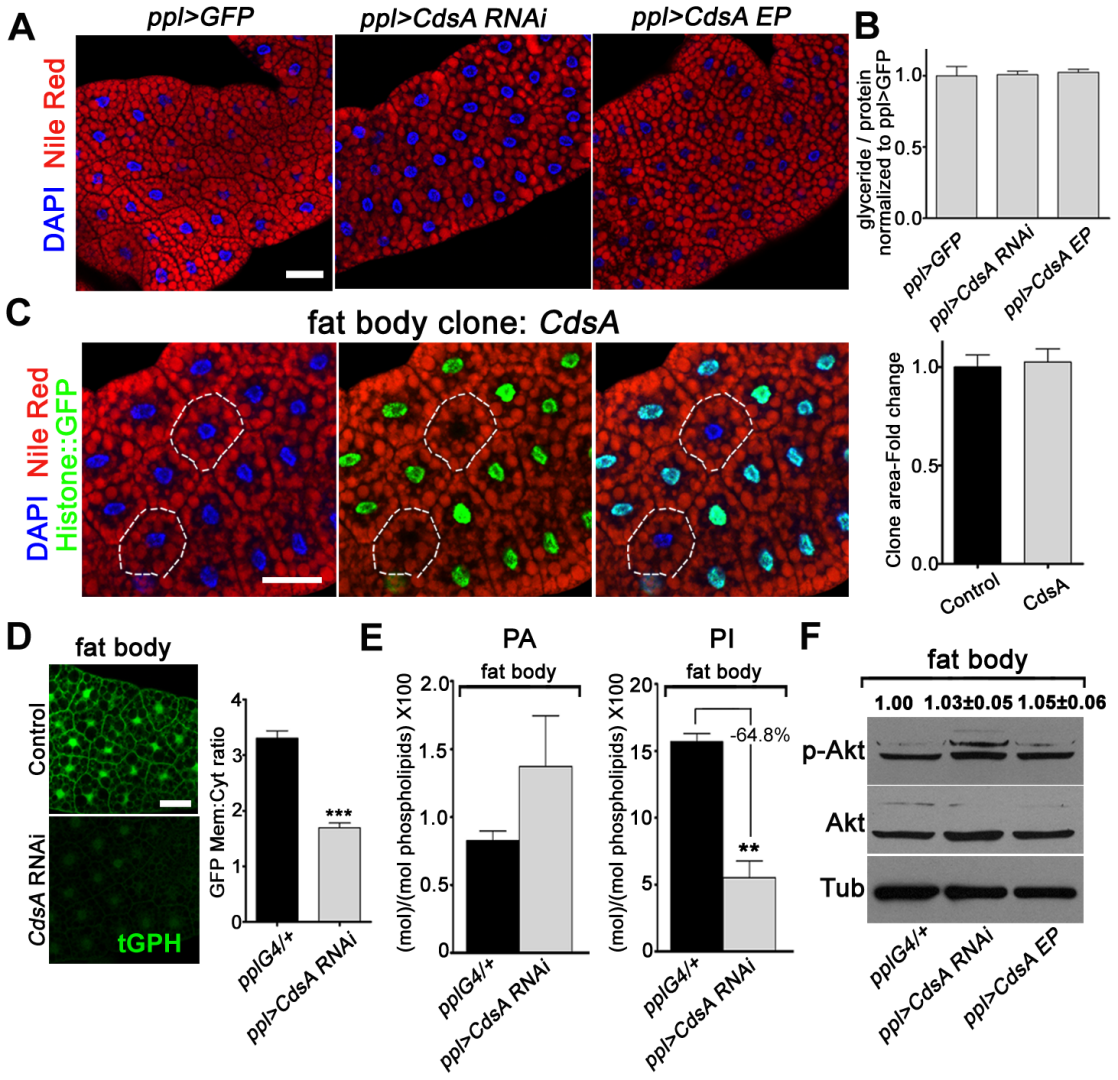
Loss of function of *CdsA* does not affect fat cell lipid storage and growth. (A) Nile red staining of wandering 3^rd^ instar larval fat body of *ppl>GFP* control, *CdsA RNAi*, and *CdsA* overexpression. Neither silencing nor overexpressing *CdsA* affects fat body lipid storage. Blue: DAPI staining for nuclei; red: Nile red staining for neutral lipids. (B) Total body fat (normalized to total protein) of wandering 3^rd^ instar male larvae of *ppl>GFP* control, *CdsA RNAi*, and *CdsA* overexpression. Glyceride assays shown here are representative experiments based on triplicate measurements with a total of ≥15 male larvae per genotype. The level of glyceride is normalized to total proteins. (C) Fat body *CdsA* mutant cells (non-GFP, dashed white circles) are normal in size and lipid content. Fat body *CdsA* mutant clones were induced by mitotic recombination 8 hr after egg laying and visualized in wandering 3^rd^ instar larvae. Blue: DAPI staining for nuclei; red: Nile red staining for neutral lipids; green, Histone::GFP. Histogram: n = 7. (D) tGPH reporter assay in fat body cells. *CdsA RNAi* leads to reduced membrane-to-cytoplasm ratio of the tGPH GFP fluorescence. Images were all taken with the same exposure time. Note that the total GFP fluorescence is also diminished. Histogram: n = 18. (E) Phospholipid levels of *ppl-Gal4* control and *ppl>CdsA RNAi* fat body from wandering 3^rd^ instar larvae. Note that the PI level in the *CdsA RNAi* fat body sample is reduced to approximately one third of that in the *Gal4* control. Assays were done in triplicate. The levels of PA and PI are normalized to total phospholipids. (F) Neither silencing nor overexpressing *CdsA* affects larval fat body Akt phosphorylation at Ser505. Total Akt and phosphorylated Akt (Ser505) levels were detected by western blotting. α-tubulin (Tub) was used as a loading control. Average and standard deviation of relative band intensity ratio of p-Akt *versus* total Akt from three replicates is indicated at the top after normalization. Western blot result for one experiment is shown here. Scale bar (A, C, D): 50 µm. Error bars (B, C, D, E) represent SEM. (*) P<0.05; (**) P<0.01; (***) P<0.001 (Student's t-test).

Besides salivary gland, we also examined the role of *CdsA* in fat body by both loss-of-function and gain-of-function analyses. To our surprise, fat body-specific *CdsA RNAi* or *CdsA* overexpression using *ppl-Gal4* or *cg-Gal4* did not result in a significant lipid storage phenotype in the fat body (Figure 5A and data not shown). Consistent with this, the total levels of glycerides are not significantly different in control, *CdsA RNAi*, and *CdsA*-overexpressing animals (Figure 5B). The fat cell size is also unchanged in *CdsA RNAi* and *CdsA*-overexpressing animals (Figure 5A). Although the RNAi efficiency was verified by Q-RT-PCR (data not shown), it is still possible that knockdown by *CdsA RNAi* is not efficient enough to cause a cell growth or fat storage phenotype in the fat body. To rule out this possibility and to obtain more definitive answers, we analyzed *CdsA* mutants. In contrast to the small size and massive neutral lipid storage of *CdsA^GS8005^* mutant salivary gland cells (Figure 2C), *CdsA^GS8005^* mutant fat body cells are normal in size and fat storage (Figure 5C), which is consistent with the RNAi result. Therefore, we concluded that fat body cell growth and neutral lipid storage are not affected by *CdsA* under normal conditions.

The lack of a neutral lipid storage phenotype in *CdsA RNAi* fat bodies suggests that PA and probably *de novo* lipogenesis from fatty acyl-CoA contribute little to final TAG content in the fat body under normal conditions. Indeed, it was reported that DAG is the major form in which lipids are transferred, via lipoproteins, from the intestine to the fat body for storage [25]. Our results are also consistent with a previous finding that unknown mechanism(s) maintain the level of fat body lipid storage within a narrow range [25].

Next, we asked why *CdsA* does not affect fat body cell growth. As we showed before, *CdsA* affects PI levels in the salivary gland and subsequently the activity of the insulin pathway (Figure 3). Is it possible that PI or insulin signaling is not important for fat body cell growth? Several previous reports involving manipulations of insulin pathway activity in fat cells clearly rule out this possibility [10], [16], [34]. Alternatively, it is possible that the PI level is not reduced in *CdsA RNAi* or mutant fat cells compared to salivary gland. We therefore examined the levels of PI and PIP3 in *CdsA RNAi* fat body cells. Similar to salivary gland, the overall tGPH reporter intensity is much lower in *CdsA RNAi* fat cell compare to control. The ratio of plasma membrane to cytosol GFP signal intensity from the tGPH reporter is reduced to around 50% of wild type in *CdsA RNAi* fat body cells (Figure 5D). By lipid content analysis, the level of PA is slightly increased in *CdsA RNAi* fat bodies (Figure 5E). However, compared to the >10-fold reduction of PI levels in the *CdsA RNAi* salivary gland, the level of PI is only reduced to 35% of wild type in the *CdsA RNAi* fat body (Figure 5E). Interestingly, the P-Akt^S505^ level is not changed in the *CdsA RNAi* fat body (Figure 5F), consistent with the cell size phenotype. These results raise the possibility that although the level of PI, and probably PIP3, is reduced by *CdsA RNAi*, the reduction is not sufficient to affect Akt phosphorylation or other compensatory mechanism(s) that exist to support fat cell growth.

### A DAG-to-PE route mediated by the choline/ethanolamine phosphotransferase Bbc may contribute to fat cell growth in *CdsA RNAi* larvae

**Figure 6:**
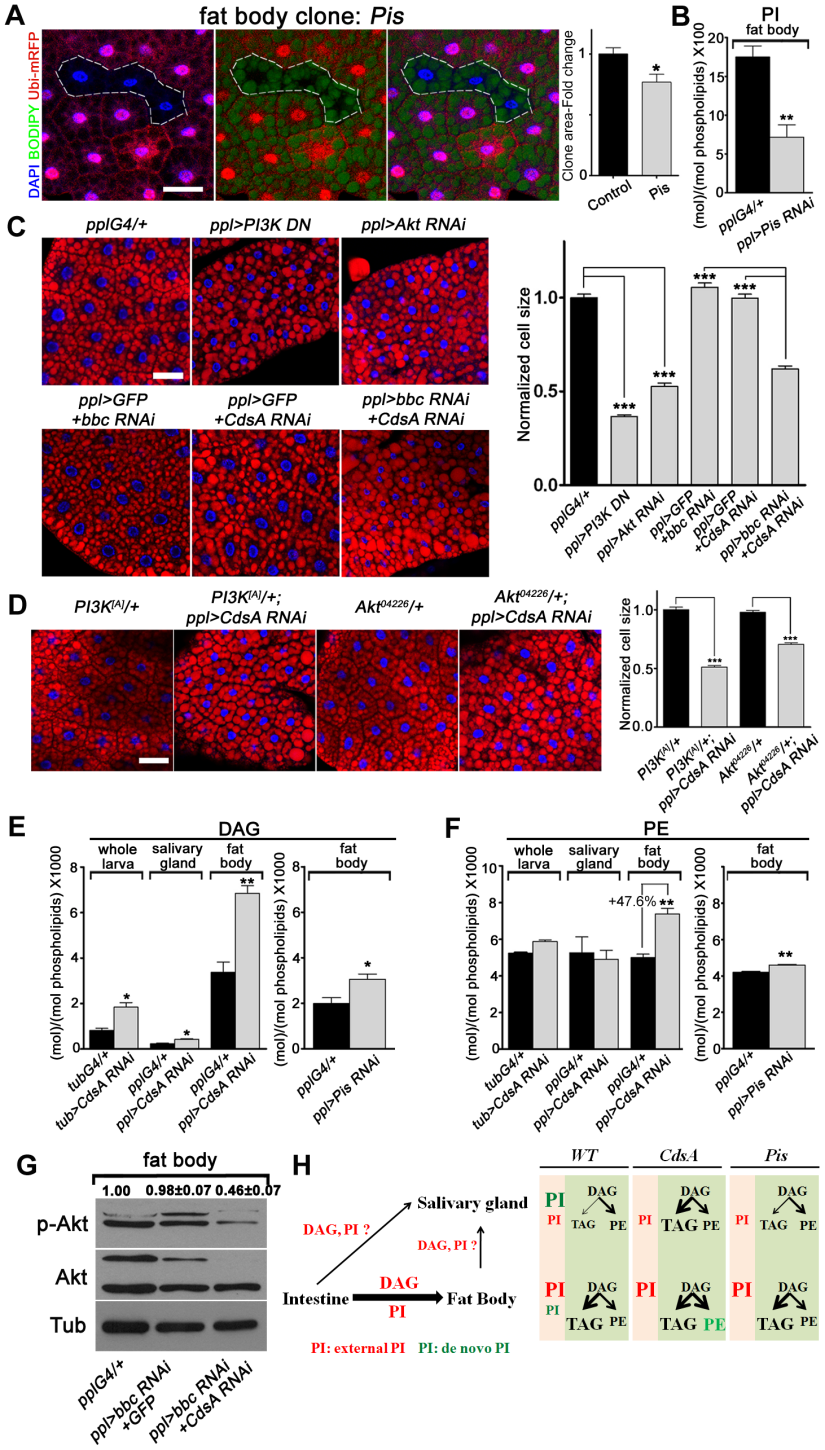
A DAG-to-PE route mediated by Bbc may support cell growth in *CdsA RNAi* fat body. (A) Fat body *Pis* mutant cells (marked by absence of RFP, dashed white area) are slightly smaller than neighboring twin-spot wild-type cells. Fat body *Pis* mutant clones were induced 8 hr after egg laying and visualized in wandering 3^rd^ instar larvae. Blue: DAPI staining; green: BODIPY staining for neutral lipids; red: Ubi-mRFP, which marks cytosol. Histogram: n = 7. (B) Total PI levels in control and *Pis RNAi* fat body. The level of PI is normalized to total phospholipids. (C) Nile red staining of fat bodies in larvae with loss of function of insulin pathway components or silencing of *CdsA* and *bbc*. Silencing *CdsA* or *bbc* alone does not affect fat body cell size, whereas reduced insulin pathway activity dramatically decreases the cell size. Double silencing of *CdsA* and *bbc* also significantly reduces fat body cell size. Histogram: n≥94 for each genotype. Blue: DAPI staining for nuclei; red: Nile red staining for neutral lipids. (D) Removing one copy of *PI3K* or *Akt* in a *ppl>CdsA RNAi* background is sufficient to reduce fat body cell size. Histogram: n≥80 for each genotype. Blue: DAPI staining for nuclei; red: Nile red staining for neutral lipids. (E, F) DAG and PE levels obtained by lipid measurements of whole larva, salivary gland or fat body of wandering 3^rd^ instar larvae of *Gal4* control, *CdsA RNAi*, *CdsA* overexpression and *Pis RNAi*. *CdsA* or *Pis* was knocked down with *ppl-Gal4* in salivary gland and fat body and *tub-Gal4* in whole larva. Assays were done in triplicate. Note that the PE level in the *CdsA RNAi* fat body sample was 47.6% higher than the *Gal4* control. The levels of DAG and PE are normalized to total phospholipids. (G) Double silencing of *CdsA* and *bbc* decreases larval fat body Akt phosphorylation at Ser505. Total Akt and phosphorylated Akt (Ser505) levels were detected by western blotting. α-tubulin (Tub) was used as a loading control. Average and standard deviation of relative band intensity ratio of p-Akt *versus* total Akt from three replicates is indicated at the top after normalization. The Western blot result from one experiment is shown here. (H) Schematic model depicting the underlying mechanisms of different phenotypes observed in *CdsA* and *Pis* mutants. In wild type, *de novo*-synthesized PI contributes most to the growth of salivary gland cells, while PI from an external source along with DAG, probably from the intestine, contributes most to the growth and fat storage of fat body cells. Both *CdsA* and *Pis* mutations lead to ∼80% growth reduction in salivary gland due to the loss of *de novo*-synthesized PI. In fat body, the loss of *de novo*-synthesized PI in *Pis* mutants results in ∼30% reduction of cell growth, while the increase in PE (marked in green) in *CdsA* mutants compensates for the loss of *de novo*-synthesized PI. Fat storage in the salivary gland of *CdsA* mutants increases dramatically. Scale bar (A, C, D): 50 µm. Error bars (A, B, C, D, E, F) represent SEM. (*) P<0.05; (**) P<0.01; (***) P<0.001 (Student's t-test).

To further explore why the fat cell growth is not affected in *CdsA* mutant, we turned to *Pis* null mutant and *Pis RNAi* again. *Pis^1^* mutant fat body cells are about 30% smaller on average than control cells (Figure 6A) and in *Pis RNAi* fat body, the PI level reduced to less than half of the control (Figure 6B). Expression of dominant negative *PI3K* or *Akt* RNAi reduces the size of fat body cells by over 50% (Figure 6C). Together, these results suggest that *de novo*-synthesized PI contributes partly to fat cell growth and PI from other origins (i.e. transported from other cells/tissues or inherited from mother cells) may contribute significantly to the growth of fat cells. If so, why is a cell growth phenotype not exhibited by *CdsA* mutant clones? To examine the *CdsA* mutant phenotype in a sensitive genetic background, we crossed *CdsA RNAi* to *PI3K* or *Akt* mutants. We found that reducing *PI3K* or *Akt* to one copy in the *CdsA RNAi* background results in a reduction in fat cell size of around 50% (Figure 6D). These dosage-sensitive interactions suggest that *CdsA RNAi* mildly impairs fat cell growth.

In addition, because *CdsA* acts one step earlier than *Pis* in PI biosynthesis (Figure 1A), it is possible that accumulation of or lack of certain metabolites compensates for the mild growth defect in *CdsA* mutants. Through lipid measurements, we found that the levels of DAG and PE are significantly increased in *CdsA RNAi* fat body samples. In fat body, the DAG level is doubled and the PE level is increased by about 50%, while in salivary gland, the PE level is unchanged (Figure 6E and 6F). In contrast, the levels of DAG and PE are only slightly increased in *Pis RNAi* fat body (Figure 6E and 6F). PEBP, a PE binding protein, has been reported to affect Akt phosphorylation, suggesting that PE may be linked to insulin signaling [35]. If the growth compensation is through the DAG-to-PE route, blocking the synthesis of PE from DAG in *CdsA RNAi* animals might lead to a more obvious fat cell growth phenotype. Choline/ethanolamine phosphotransferase (Cept) Bbc is a key enzyme in *Drosophila* that converts DAG to PE (Figure 1A) [36]. While *bbc RNAi* did not affect the P-Akt^S505^ level (Figure 6G) and did not obviously affect the fat cell size (Figure 6C), *CdsA* and *bbc* double RNAi exhibits a synergistic effect: the fat cell size is drastically decreased to a level comparable to *Akt* or *PI3K RNAi* (Figure 6C). Moreover, the fat body P-Akt^S505^ level is greatly reduced by *CdsA* and *bbc* double RNAi (Figure 6G). In contrast, the salivary gland cell size of *CdsA* and *bbc* double RNAi is comparable to *CdsA* single RNAi (Figure S3). Together, these results suggest that the elevated conversion of DAG to PE mediated by the choline/ethanolamine phosphotransferase Bbc may contribute to fat cell growth in *CdsA RNAi* larvae.

## Discussion

The regulation of cell growth in terms of both cell size and cell number has been studied intensively, and is known to involve many key core signaling pathways such as the insulin pathway, the Hippo pathway, and the mTOR pathway, as well as cross-talk between these pathways [5], [7], [37]. The downstream targets that execute and coordinate various specific aspects of cell growth are just starting to be elucidated. In this study we have revealed the intrinsic connections between CDP-diacylglycerol synthetase, PI metabolism, and insulin pathway activity in coordinating cell growth and neutral lipid storage.

Lipid storage is a basic function of a cell. Within an organ, glycerol and fatty acyl-CoA is the precursor of all phospholipids, which are mainly utilized within the cell, and glycerolipids, which are largely destined for neutral lipid storage. Therefore, under normal developmental conditions when resources are not unlimited, a balance must be achieved between cell growth and storage (fast growth and less storage, or slow growth and increased storage). Moreover, at the systematic level, the tissue specificity of insulin action and the lipid flow between different tissues make this balance more complicated [38]. For non-adipose tissues, fast cell growth and low levels of storage are beneficial for maximizing growth potential. However, the balance encounters a problem in adipose tissue because both growth and storage are required. Several predictions can be made here. First, the key enzymes that act at the branch points of phospholipid and TAG synthesis are good candidates for controlling the balance between growth and storage. Second, the factors that control the difference in balance regulation between adipose tissue and non-adipose tissue likely sit after the regulatory points. Alternatively, adipose tissue may have two developmental phases, a growing phase and a fat storage phase. The timing of the switch between these two phases would determine the final size and storage capacity of adipose tissue. Third, excess nutrients may saturate the balance mechanism, leading to overload in both directions. Indeed, our results depict a scenario in which CDP-diacylglycerol synthetase coordinates cell growth and fat storage by partitioning the flow of fatty acyl-CoA between PI synthesis and TAG synthesis. Moreover, our results reveal different contributions of *de novo*-synthesized PI and PI derived from external sources to the growth of fat cells and salivary gland cells. *De novo*-synthesized PI contributes most to the growth of salivary gland cells, while fat body cell growth is mainly stimulated by externally-derived PI (Figure 6H). Therefore, the role of *CdsA* in fat cell growth is only revealed under conditions in which growth is mildly compromised. Besides that, an external supply of DAG, most likely from the intestine, may bypass the CdsA-gated coordination, allowing adipose tissue (fat body) cells to both grow large and store fat (Figure 6H). Consistent with this, it was previously reported that intestine-derived lipids, including DAG, are mainly transported to the fat body via lipoproteins, while relatively low levels of lipids are transported to non-adipose tissues either directly from the intestine or indirectly via the fat body [25], [39].

By responding to environmental and nutritional cues, the insulin pathway regulates cell growth [5], [9]. Through genetic analyses, GFP reporter assays, and lipidomics, we have presented evidence for a positive feedback loop between the insulin pathway and CdsA levels (Figure 4G). In this loop, lowering insulin pathway activity reduces *CdsA* transcription, leading to less PI production, which further decreases insulin pathway activity. There are numerous previous findings of FOXO as a transcriptional activator [20], [33], [40], but dFOXO appears to be a repressor of *CdsA* transcription. Microarray and ChIP studies have identified many other targets of FOXO repression [41]. At this point, we do not know whether dFOXO represses *CdsA* directly or indirectly. The widespread cell growth and fat storage phenotype caused by *CdsA RNAi* suggests that the insulin pathway-CdsA feedback loop operates widely in various tissues. In addition, it shows that the fat storage capacity of most non-adipose tissues is enormous.

The insulin pathway-CdsA feedback regulation allows cells to take full advantage of the environmental conditions to grow fast and stop growth quickly when necessary. For example, in a nutrient-rich environment, animals that can grow quickly have a tremendous competitive advantage over slow-growing ones. In nutrient-poor conditions, the ability to quickly convert growth to fat storage is beneficial for animals to survive through harsh times. This is not the first time that feedback regulation of the insulin pathway has been reported [33], suggesting that it might be a common mechanism to augment pathway sensitivity and ensure a rapid response to changes in nutrient conditions. Regulation of the insulin pathway by lipids is already known to occur at the points of PIP to PIP2 and PIP2 to PIP3 conversion. Compared to regulation by PI3K and PTEN, *CdsA* provides a new layer of control at the level of available lipids. Together with other pathways regulated by available nutrients/small molecules, such as the mTOR pathway, which responds to amino acid levels, and the AMPK pathway, which responds to ATP levels [42], [43], this new regulatory mechanism helps cells to cope with diverse environmental changes.

The insulin-CdsA feedback loop is important during cell development. When cells reach the homeostasis phase, the growth-favored loop must be switched towards fat storage. In the future, it will be interesting to determine what triggers the switch. In addition, the existence of the positive feedback loop suggests that excess nutrients will overload the fatty acyl-CoA partitioning mechanism and disrupt the balance. Therefore, impairing the insulin-CdsA feedback loop by genetic mutations or environmental conditions may cause an imbalance in fat storage, resulting in metabolic diseases such as obesity and related disorders. Re-establishing the feedback loop could be a way of treating these disorders.

## Materials and Methods

### *Drosophila* stocks and husbandry

*Drosophila* stocks were maintained in standard cornmeal food. *w^1118^* was used as wild type. All RNAi stocks were obtained from the VDRC or NIG RNAi stock centers. *ppl-Gal4* was generously provided by Dr. Pierre Leopold. The expression pattern of *ppl-Gal4* was confirmed with *UAS-GFP*. Other stocks used were: *tub-Gal4*, *y^1^ w^67c23^; P{EPgy2}CdsA^EY08412^*, *y^1^ w^67c23^; P{GSV3}GS8005/TM3 Sb^1^ Ser^1^*, y^1^ w*; P{w[+mC] = Dp110[D954A]}2, *y^1^ w^1118^; P{w[+mC] = UAS-Akt1.Exel}2*, *P{w[+mC] = Dp110-CAAX}1*, *dFOXO^21^*, *dFOXO^25^*, *w^1118^; P{w[+mC] = tGPH}2; Sb^1^/TM3 Ser^1^*, *Pis^1^/FM7a; P{w[+mC] = hs-Pis.MYC}3/TM2*, w*; Df(3R)Pi3K92E[A] H[A] Pi3K92E[A], *P{ry[+7.2] = HBS}3/TM6B Tb^1^*, *ry^506^ P{ry[+t7.2] = PZ}Akt1[04226]/TM3 ry^RK^ Sb^1^ Ser^1^*, *P{w[+mC] = Ubi-mRFP.nls}1*, w* P{ry[+t7.2] = hsFLP}122 P{ry[+t7.2] = neoFRT}19A, *hsflp122;sp/Cyo;FRT2A histone:GFP/TM6B, ry^506^ P{HZ}CdsA^1^*.

### RNAi screen and genetic analysis

*ppl-Gal4* virgin females were crossed to males harboring *UAS-RNAi* inserts to knock down target gene expression in the larval salivary gland. Wandering 3^rd^ instar larvae harboring both the *Gal4* and the *UAS-RNAi* transgene were dissected and mounted on glass slides with PBS. For visualization, specimens were examined with a Zeiss DIC microscope. For *GAL4/UAS* experiments, flies were grown at 29°C to allow for maximal GAL4 activity. For tissue or clonal analysis, unless stated otherwise, all larvae were dissected at the wandering 3^rd^ instar stage. Salivary gland clones were generated by heat shock at 37°C for 1 hr immediately after collection of eggs for 4 hr. Fat body clones were induced 8 hr after egg laying for 1 hr at 37°C.

### Staining and microscopy

After crossing *ppl-Gal4* with RNAi lines, wandering 3^rd^ instar larval progeny were dissected and stained with Nile red or Bodipy, which are fluorescent dyes that specifically mark neutral lipids. For lipid droplet staining, larvae were dissected in PBS and fixed in 4% paraformaldehyde for 30 min at room temperature. Tissues were then rinsed twice with PBS, incubated for 30 min in a 1∶2500 dilution with PBS of 0.5 mg/ml Nile red (Sigma), and then rinsed twice with distilled water. Stained samples were mounted in 80% glycerol for photo-taking. For β-galactosidase staining, dissected tissues were fixed for 2 min in 0.5% glutaraldehyde on ice. The tissues were washed three times for 10 min each in PBS containing 0.3% Triton X-100 (PBT) and then placed in X-gal staining solution (0.2% X-gal, 1 mM MgCl_2_, 5 mM K_4_[Fe(CN)_6_], 5 mM K_3_[Fe(CN)_6_]) overnight at 37°C. All images were taken using a Nikon confocal scope or Zeiss fluorescent scope. Quantification of mutant clone areas was performed by calculating the size of the area occupied by mutant clones versus wild-type cells. Quantification of the size of whole salivary gland, brain, and wing disc or the size of fat body cells was performed by measuring the area occupied by these organs/cells. To quantify lipid storage in salivary glands, the Nile red positive areas of salivary glands per genotype were measured and normalized to the whole cell area as previously described [25]. The average lipid storage in salivary glands of *CdsA RNAi* was set as 1. For tGPH signal quantification, membrane-to-cytoplasmic GFP ratios were calculated from measurements of mean pixel intensities within equal areas of membrane versus cytoplasm. All measurements were done using Nikon Br analysis software.

### Quantitative RT-PCR

Total RNA was isolated using an RNeasy kit (Qiagen) according to the manufacturer's protocol. To quantify *CdsA* transcript levels in *tub>CdsA RNAi* and *tub/+* controls, three groups of 5 larvae per genotype were collected at the wandering 3^rd^ instar larval stage. To quantify *CdsA* transcript levels in different tissues such as salivary gland, fat body, brain, and gut, three groups of 30 tissues per genotype were dissected and collected from wandering 3^rd^ instar larvae. To quantify *CdsA* transcript levels in larval salivary gland with loss of function or overexpression of insulin pathway components, salivary glands were dissected and collected in three groups of 50 pairs per genotype from wandering 3^rd^ instar larvae. For each sample, 3 µg total RNA was used to synthesize single-stranded cDNA using a SuperScript II reverse transcriptase kit (Invitrogen). Quantitative RT-PCR (qRT-PCR) experiments (Agilent Stratagene Mx3000P system, SYBR Green PCR mix) were performed using specific primer pairs (5′ GCAATGATGTCATGGCGTAC 3′ and 5′ AAATCCAGAGGCAAAGAAGC 3′). The level of each transcript was normalized to *rp49* in the same sample. All qRT-PCR experiments were repeated at least three times.

### Western blot and quantification

Whole larva, salivary gland, and fat body extracts were prepared by dissecting wandering 3^rd^ instar larvae in groups of 6, 120, and 30 larvae/tissues respectively. Extracts were lysed by homogenizing equal masses of control or *RNAi* larval tissues on ice in 200 µl of ice-cold 1% SDS lysis buffer (1% SDS, 40 mM Tris-Cl PH7.45, 1 mM PMSF, 1 tablet Protease inhibitor, PH7-8). For western blotting, 30 µg of protein sample were loaded, blotted, and detected with the following antibodies: rabbit anti-Akt (Cell Signaling, diluted at 1∶1000), rabbit anti-phospho-Akt (Ser473) (Cell Signaling, diluted at 1∶1000), and rabbit anti-α-tubulin (Abcam, diluted at 1∶4000). Quantification of band intensities was done using Image J (National Institutes of Health) software.

### PIP3 ELISA assay

The extraction and measurement of PIP3 were performed using the PIP3 Mass ELISA Kit (Echelon), according to the manufacturer's instructions. For extraction of PIP3, four groups of 20 larvae per genotype were collected at the wandering 3^rd^ instar larval stage. The level of PIP3 was normalized to larval dry mass in each group. The PIP3 ELISA assay was repeated four times.

### Lipid analysis and quantification of total glycerides

Lipids were extracted from salivary glands of 50 larvae, fat bodies of 50 larvae, 12 whole larvae or 6 fat body-removed larvae carcass (three sets of samples per genotype). An Agilent high performance liquid chromatography (HPLC) 1260 system coupled with an Applied Biosystem Triple Quadrupole/Ion Trap mass spectrometer (4500Qtrap) was used to quantify individual lipids. Polar lipids were analyzed using multiple reaction monitoring (MRM) scans [44]. Separation of individual polar lipids was carried out using a Phenomenex Luna 3u silica column (i.d. 150×2.0 mm). Individual lipid species were quantified by referencing to spiked internal standards. PC-14:0/14:0, PE-14:0/14:0, PS-34:1/d_31_, PA-17:0/17:0, PG-14:0/14:0 and PI-34:1/d_31_ were obtained from Avanti Polar Lipids. Neutral lipids were analyzed using a sensitive HPLC/ESI/MS method modified from a previous method [45]. DAGs were quantified using 4ME 16∶0 Diether DG (Avanti) as an internal standard. Quantitative analysis of PIP2 was carried out as described [46] using a Dionex Ion Chromatography 5000 system. Lipid extracts were deacylated by incubation with 0.5 ml of methylamine reagent [MeOH/40% methylamine in water/1-butanol/water (47∶36∶9∶8)] at 50°C for 45 min. The aqueous phase was dried, resuspended in 0.5 ml of 1-butanol/petroleum ether/ethyl formate (20∶40∶1), and extracted twice with an equal volume of water. Aqueous extracts were dried, resuspended in water, and subjected to anion-exchange HPLC on an Ionpac AS11-HC column. Lipid levels were calculated using deacylated anionic phospholipids as standards.

Total body glycerides were measured by homogenizing six groups of 5 male larvae per genotype in 100 µl PBS containing 0.05% Tween 20 (PBST). The homogenate was immediately heat-treated at 70°C for 10 min. Then, 20 µl samples were incubated with 200 µl Triglyceride Reagent (Triglycerides Kit, ZhongShengBeiKong) for 10 min at 37°C and the absorbance at 540 nm was measured using a spectrophotometer. The total protein content of the same samples was measured by Bradford assay (Sigma). Glyceride levels were normalized to total protein amounts in each sample.

## Supporting Information

Figure S1 *CdsA* is widely expressed in various tissues. (A) *ppl>CdsA RNAi* with an independent *UAS-RNAi* line from VDRC has a similar small salivary gland size and ectopic fat storage phenotype. (B) Relative expression levels of *CdsA* transcripts in various tissues were quantified by qRT-PCR. Measurements were made in triplicate. (C) *CdsA* expression is indicated by staining for β-galactosidase expression in a Lac-Z enhancer trap line in the *CdsA* genomic locus. Scale bar (A, C): 50 µm.
(TIF)

Figure S2 Quantification of lipid storage in Fig. 4. Histogram (A, B): n≥8 for each genotype. Error bars (A, B) represent SEM. (*) P<0.05; (**) P<0.01; (***) P<0.001 (Student's t-test).
(TIF)

Figure S3 Double knockdown *CdsA* and *bbc* didn't enhance the salivary gland size phenotype of *CdsA* single *RNAi*. (A) The salivary gland size reduction of *CdsA* and *bbc* double *RNAi* is comparable to *CdsA RNAi* alone. Blue: DAPI staining for nuclei; red: Nile red staining for neutral lipids. (B) Relative sizes of salivary glands were quantified. n≥8 for each genotype. Error bars represent SEM.
(TIF)
