# Supplementary figures and images for: CDP-Diacylglycerol Synthetase Coordinates Cell Growth and Fat Storage through Phosphatidylinositol Metabolism and the Insulin Pathway
Source: PLoS Genet. 2014 Mar 6;10(3):e1004172. doi: 10.1371/journal.pgen.1004172 (PMC3945474; doi:10.1371/journal.pgen.1004172)

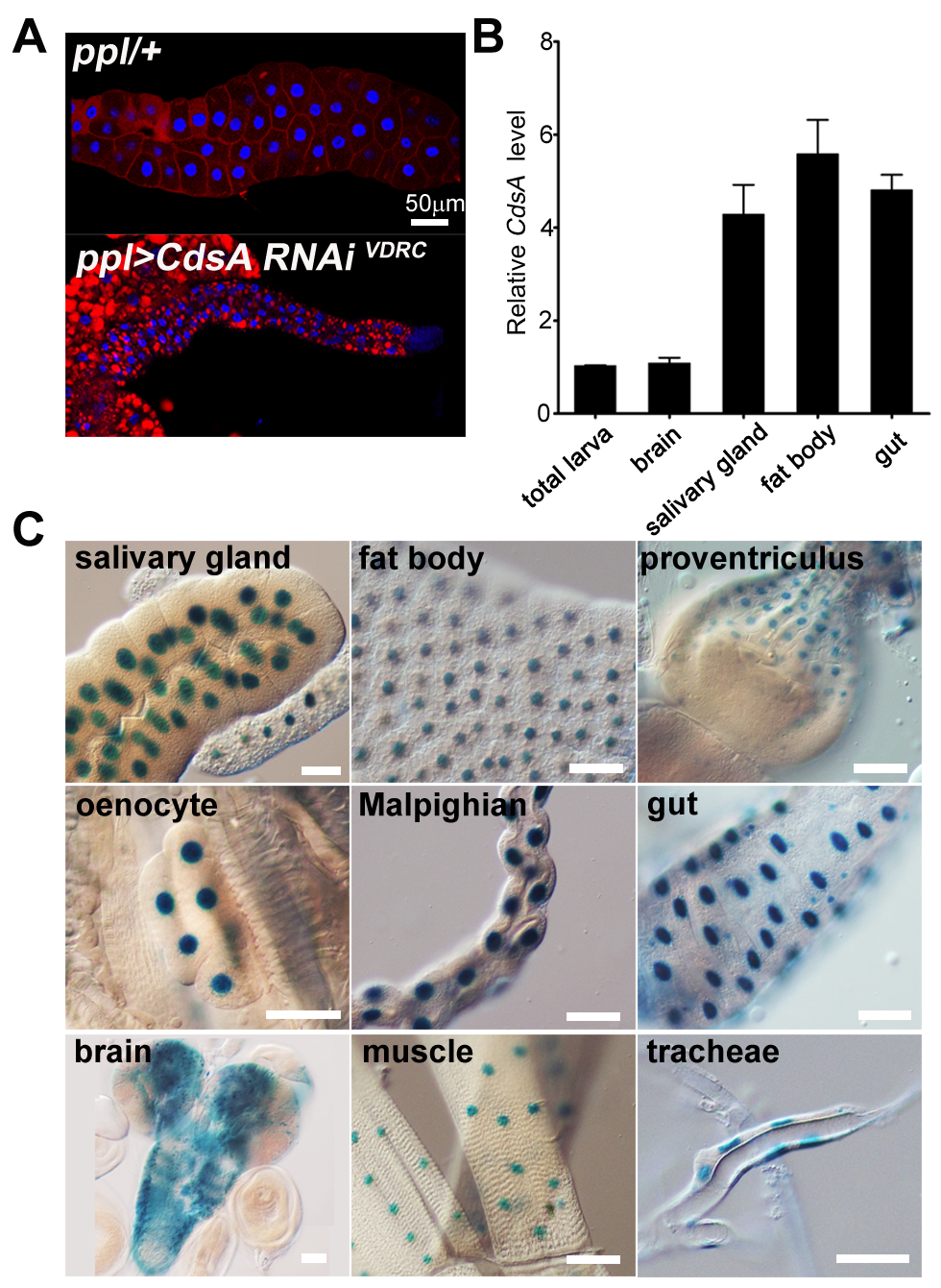

Supplement: Figure S1 — CdsA is widely expressed in various tissues. (A) ppl>CdsA RNAi with an independent UAS-RNAi line from VDRC has a similar small salivary gland size and ectopic fat storage phenotype. (B) Relative expression levels of CdsA transcripts in various tissues were quantified by qRT-PCR. Measurements were made in triplicate. (C) CdsA expression is indicated by staining for β-galactosidase expression in a Lac-Z enhancer trap line in the CdsA genomic locus. Scale bar (A, C): 50 µm. (TIF) [file pgen.1004172.s001.tif]

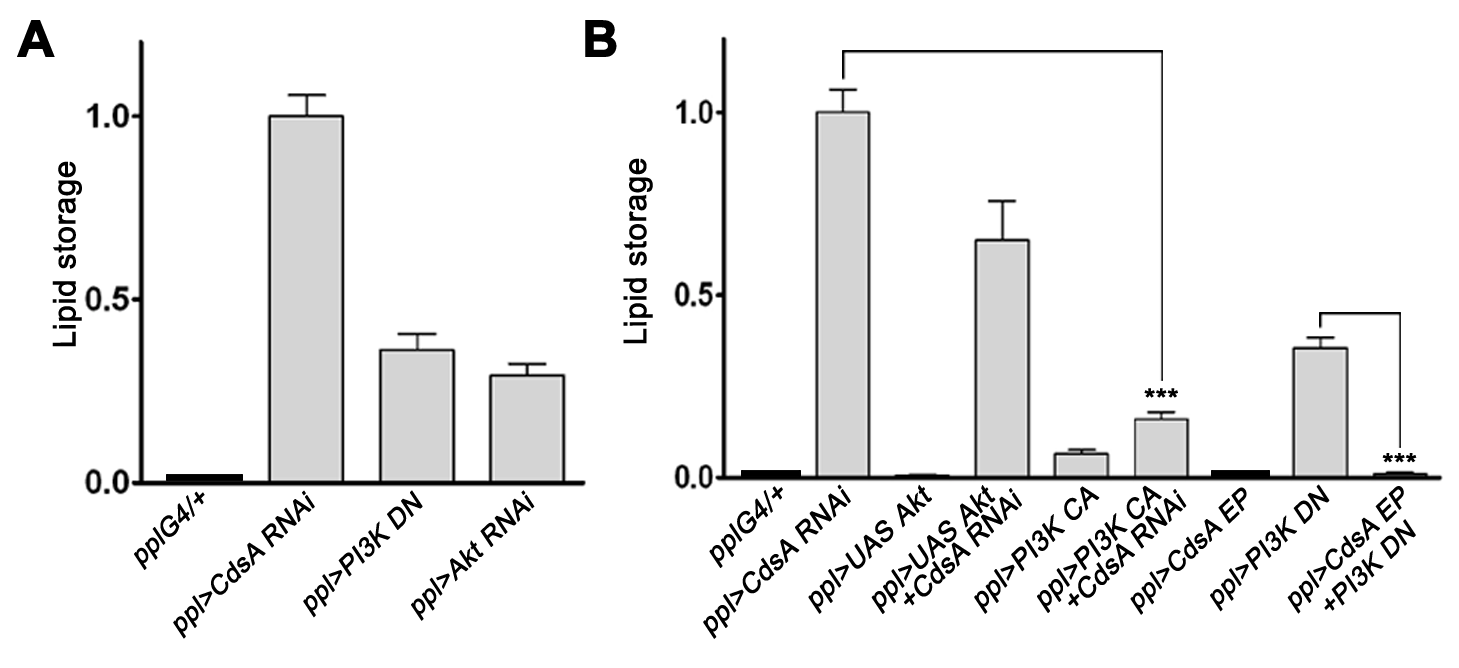

Supplement: Figure S2 — Quantification of lipid storage in Fig. 4. Histogram (A, B): n≥8 for each genotype. Error bars (A, B) represent SEM. (*) P<0.05; (**) P<0.01; (***) P<0.001 (Student's t-test). (TIF) [file pgen.1004172.s002.tif]

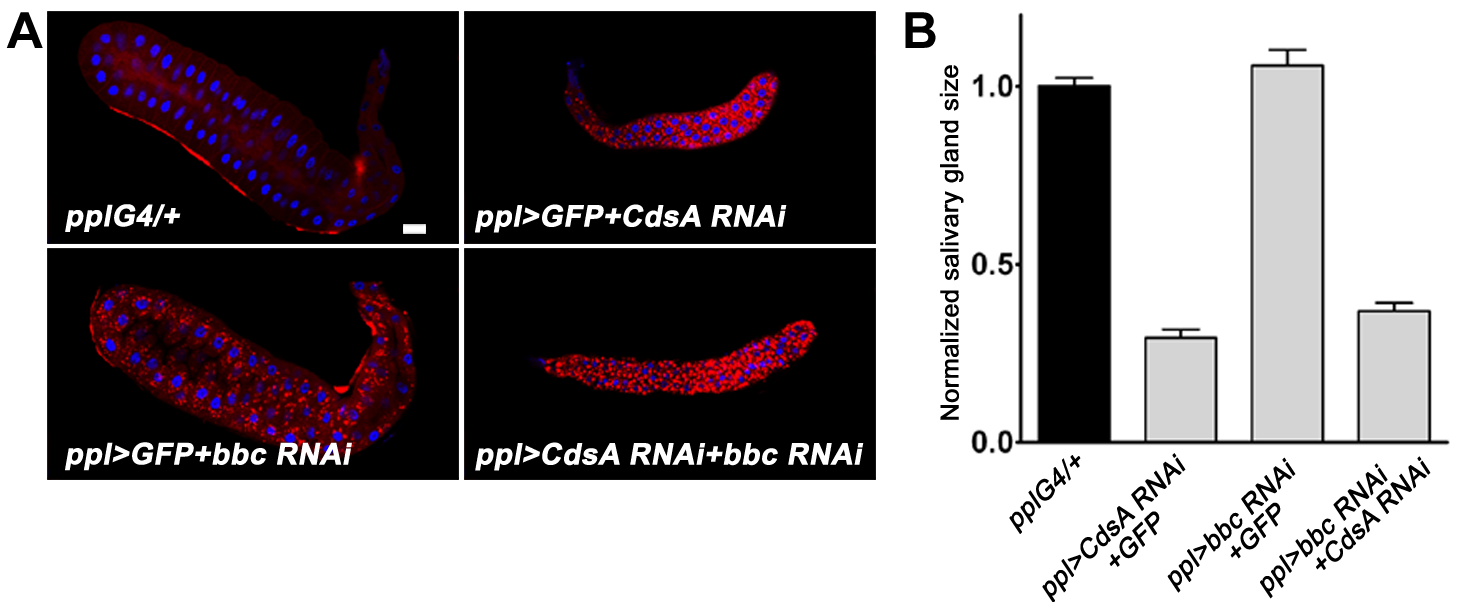

Supplement: Figure S3 — Double knockdown CdsA and bbc didn't enhance the salivary gland size phenotype of CdsA single RNAi. (A) The salivary gland size reduction of CdsA and bbc double RNAi is comparable to CdsA RNAi alone. Blue: DAPI staining for nuclei; red: Nile red staining for neutral lipids. (B) Relative sizes of salivary glands were quantified. n≥8 for each genotype. Error bars represent SEM. (TIF) [file pgen.1004172.s003.tif]
